# Combinatorial Biosynthesis of Novel Multi-Hydroxy Carotenoids in the Red Yeast *Xanthophyllomyces dendrorhous*

**DOI:** 10.3390/jof3010009

**Published:** 2017-02-22

**Authors:** Hendrik Pollmann, Jürgen Breitenbach, Hendrik Wolff, Helge B. Bode, Gerhard Sandmann

**Affiliations:** 1 Biosynthesis Group, Molecular Biosciences, Fachbereich Biowissenschaften, Goethe Universität Frankfurt, Frankfurt am Main 60438, Germany; pollmann@bio.uni-frankfurt.de (H.P.); Breitenbach@em.uni-frankfurt.de (J.B.); 2 Merck Stiftungsprofessur für Molekulare Biotechnologie, Fachbereich Biowissenschaften, Goethe Universität Frankfurt, Frankfurt am Main 60438, Germany; wolff@bio.uni-frankfurt.de (H.W.); h.bode@bio.uni-frankfurt.de (H.B.B.); 3 Buchmann Institute for Molecular Life Sciences (BMLS), Goethe Universität Frankfurt, Max-von-Laue-Strasse 15, Frankfurt am Main 60438, Germany

**Keywords:** carotenoid biosynthesis, 4,4’-dihydroxy-nostoxanthin, 4,4’-diketo-nostoxanthin, genetic engineering, HPLC separation, MS-MS spectra, *Xanthophyllomyces dendrorhous*

## Abstract

The red yeast *Xanthophyllomyces dendrorhous* is an established platform for the synthesis of carotenoids. It was used for the generation of novel multi oxygenated carotenoid structures. This was achieved by a combinatorial approach starting with the selection of a β-carotene accumulating mutant, stepwise pathway engineering by integration of three microbial genes into the genome and finally the chemical reduction of the resulting 4,4’-diketo-nostoxanthin (2,3,2’,3’-tetrahydroxy-4,4’-diketo-β-carotene) and 4-keto-nostoxanthin (2,3,2’,3’-tetrahydroxy-4-monoketo-β-carotene). Both keto carotenoids and the resulting 4,4’-dihydroxy-nostoxanthin (2,3,4,2’,3’,4’-hexahydroxy-β-carotene) and 4-hydroxy-nostoxanthin (2,3,4,2’3’-pentahydroxy-β-carotene) were separated by high-performance liquid chromatography (HPLC) and analyzed by mass spectrometry. Their molecular masses and fragmentation patterns allowed the unequivocal identification of all four carotenoids.

## 1. Introduction

*Xanthophyllomyces dendrorhous* (with *Phaffia rhodozyma* as its anamorphic state) is a basidiomycetous red yeast that accumulates astaxanthin [1], which is a unique feature among fungi [2]. This carotenoid is formed via the mevalonate pathway starting with a condensation of two molecules of geranylgeranyl pyrophosphate, a 4-step desaturation, cyclization and a final 4-ketolation plus 3-hydroxylation [3,4]. In contrast to other organisms, only three genes are involved in the whole pathway from phytoene to astaxanthin, which facilitates genetic modification of carotenoid biosynthesis in *X. dendrorhous.* This yeast has the potential to be engineered as a cell-factory for the production of industrially valuable carotenoids [5]. Tools and techniques for genetic manipulations of *X. dendrorhous* are available [6], including several integrative transformation plasmids based on four different selection markers [7,8]. It is also advantageous for the development of high-yield carotenoid producers since a carotenoid pathway that can be manipulated is already established, a carotenoid storage system exists and a very active acetyl-CoA metabolism can be utilized. The published genomic sequence of *X. dendrorhous* CBS6938 [9] is also very helpful for genetic engineering of the carotenoid pathway.

The potential of *X. dendrorhous* for the production of economically interesting carotenoids like astaxanthin for feed and zeaxanthin as a nutraceutical for eye care has been demonstrated. The highest production of astaxanthin was reached by combining classical mutagenesis with genetic pathway engineering [7]. Starting from a high-yield astaxanthin mutant, genes of three limiting enzymes were over-expressed, enhancing metabolite flow toward carotenoid biosynthesis and into the astaxanthin pathway [7].

Zeaxanthin is another carotenoid of interest as a nutraceutical, which is important for protection of our vision. For the engineering of a zeaxanthin producing strain, a mutant with inactive astaxanthin synthase accumulating β-carotene [10,11] was used to extend the carotenoid pathway to zeaxanthin by expression of a bacterial β-carotene hydroxylase gene and engineering of an enhanced metabolite flow into the carotenoid pathway [12].

Hydroxylated carotenoids such as the dihydroxy compound zeaxanthin [13], tetrahydroxy-β-carotene derivative nostoxanthin [14] and various other hydroxylated acyclic carotenoids [15] have been generated in *Escherichia coli* by combination of carotenogenic genes from different organisms. It has been shown that not only zeaxanthin but also several other hydroxyl derivatives have superior antioxidative activity. In our engineering approach with *X. dendrorhous*, we utilized β-carotene mutants to integrate hydroxylase and ketolase genes from bacteria and algae and finally chemically reduced the resulting tetrahydroxy-monoketo and tetrahydroxy-diketo products to a pentahydroxy and a hexahydroxy β-carotene derivative, respectively.

## 2. Materials and Methods

### 2.1. Strains and Cultivation

The β-carotene accumulation mutant of *X. dendrorhous* strain PR1-104 was generated by ethyl-methane sulphonate mutagenesis and selection for carotenoid content [10]. This mutant and transformants were grown as shaking cultures (50 mL in 500 mL baffled Erlenmeyer flasks, 180 rpm) over 7 days in YM medium (0.3% yeast extract, 0.3% malt extract, 0.5% peptone, 1.0% glucose) with white light illumination. For selection of transformants on agar plates, geneticin (G418 sulfate, 100 µg/mL), hygromycin (60 µg/mL) or nourseothricin (30 µg/mL) were added. *Escherichia coli* strains DH5α and JM110 used for genetic manipulations and generation of carotenoid standards were grown in LB medium containing ampicillin (100 µg/mL) and chloramphenicol (34 µg/mL) according to the plasmids involved.

### 2.2. Plasmid Construction and Transformantion of X. dendrorhous, Combinatorial Biosynthesis of Carotenoid Standards in E. coli

The integrative plasmids pPR2TNo-crtZo [12] and pPRcDNA1bkt830 [11] were previously described. Details on the origin of genes and the primers for the amplification of the *X. dendrorhous* transformation plasmids are shown in Table 1. For the construction of plasmid pPR2TNH-crtG, the *crtG* gene from plasmid pUC-Bre-O11 [16] was amplified, which also generated an *Eco*RI and *Xho*I restriction site (Table 1), and was cloned into the *Xcm*I site of the *E. coli* plasmid pMon38201 by its a-overhang [17]. From there, the *crtG* gene was cut out with *Eco*RI and *Xho*I and ligated into pUC8ΔEcoRI-HNNH [7] for fusion with the promoter and terminator of the glyceraldehyde phosphate dehydrogenase gene from *X. dendrorhous*. Then, the whole cassette was cut out by restriction with *Hin*dIII and ligated into the *Hin*dIII site of pPR2TNH [7], yielding pPR2TNH-crtG. Transformation of *X. dendrorhous* was performed by electroporation as described in Visser et al. [6] with 10 μg plasmid DNA. After phenol-chloroform purification, plasmid DNA was linearized by digestion with *Sfi*I.

Carotenoid standards were generated in *E. coli* by combinatorial transformation of plasmids pACCAR25ΔcrtX [18] plus pUC-Bre-O11 [16] for nostoxanthin, caloxanthin and zeaxanthin, pACCAR25ΔcrtX [18] plus pCRBKT [19] for canthaxanthin, echinenone and β-carotene and pACCAR25ΔcrtX plus pPEU30crtO [20] for the synthesis of 4-keto-zeaxanthin.

### 2.3. Carotenoid Extraction, Purification and Chemical Derivatization

For carotenoid extraction, 20 mg of freeze-dried *X. dendrorhous* cells were mixed with 500 µL glass beads (0.25–0.5 mm), 675 µL methanol and 75 µL of a 60% KOH solution for saponification and broken in a cell mill (Retsch MM 400) for 8 min at a frequency of 30 per second and heated for 20 min at 60 °C. Samples containing keto carotenoid were extracted without addition of KOH. After partitioning of the carotenoid extract into 50% diethyl ether in petrol (bp 40–60 °C), the upper phase was collected and dried in a stream of nitrogen. Carotenoids were quantified from three independently grown cultures.

For the purification of multi-hydroxy keto carotenoids, the extract of PR1-104-ZGbkt was fractionated by TLC on activated silica plates developed with toluene/ethyl acetate/methanol (65:35:5, by volume). The bands with an *R_f_* value of 0.20 and 0.25 were collected and extracted with acetone.

The reduction of carotenoid ketones dissolved in ethanol to the corresponding alcohol was performed according to Eugster [22] with NaBH_4_. The reaction mixture was transferred to 65% diethyl ether in petrol (bp 40–60 °C) and the upper phase collected and dried in stream of nitrogen. The same procedure was applied to canthaxanthin, yielding 4,4’-diketo-β-carotene together with 4-HO-4’-keto-β-carotene and to echinenone yielding 4-HO-β-carotene.

### 2.4. HPLC and HR-ESI-MS Analysis

HPLC analysis was performed on three different HPLC systems. For separation of the non-ketolated multi-hydroxy carotenoids, system I with a 15 × 0.4 cm Nucleosil 100 C18, 3 µm column and acetonitrile (ACN)/methanol/2-propanol (85:10:5, by volume) plus 3% H_2_O [23] was used as mobile phase with a flow rate of 0.8 mL/min at 10 °C. System II was employed for the separation of the hydroxy-keto carotenoids and their reduction products on a 25 cm C30 RP, 3 µm column (YMC, Wilmington, NC, USA) with a mobile phase of 3% methyl tertiary-butyl ether in methanol for 48 min followed by an increase to 20% with a flow rate of 0.8 mL/min at 20 °C. This system was also used for the quantification of the keto derivatives. HR-ESI-MS analysis was carried out in system III on a 2.1 mm × 50 mm ACQUITY UPLC BEH C18, 1.7 µm column. A binary gradient was applied with ACN (+0.1% formic acid) and H_2_O (+0.1% formic acid) for 12 min in the following steps: 0–2 min 5% ACN , 2–2.5 min 40% ACN, 2.5–4 min 40% ACN, 4–14 min 40%–95% at a flow rate of 0.4 mL/min at 40 °C. This HPLC system was coupled to an Impact II QTOF (Bruker) mass spectrometer using Na-formiate as an internal calibration standard [24]. Carotenoids were detected in a positive ion mode with scanning range from 100–1200 *m*/*z*. Optical spectra were recorded online with a photodiode array detector 994 (Waters, Milford, CT, USA). Carotenoid standards for identification were generated in *E. coli* by the combination of different *crt* genes as previously described [25].

## 3. Results

**Figure 1:**
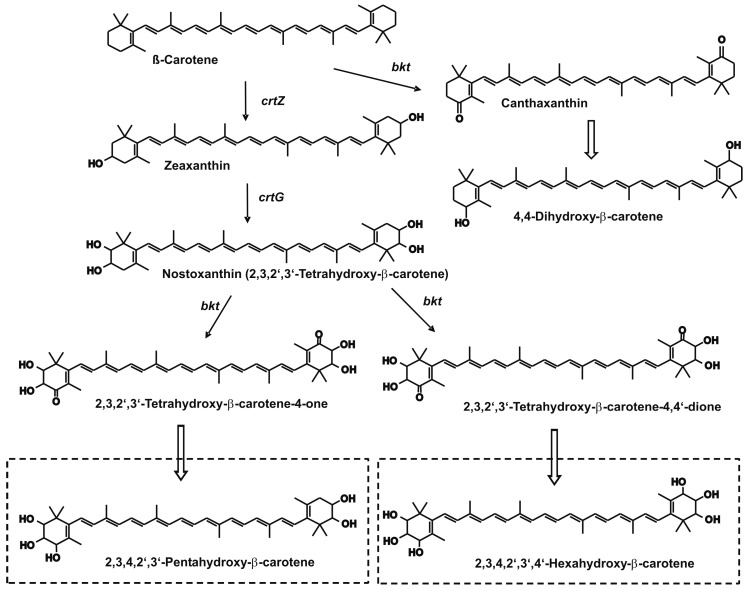
Pathway construction by genetic engineering of *Xanthophyllomyces dendrorhous* for the synthesis of multi-oxygenated β-carotene derivatives. Open arrows indicate chemical reduction. Novel carotenoid structures are boxed.

**Figure 2:**
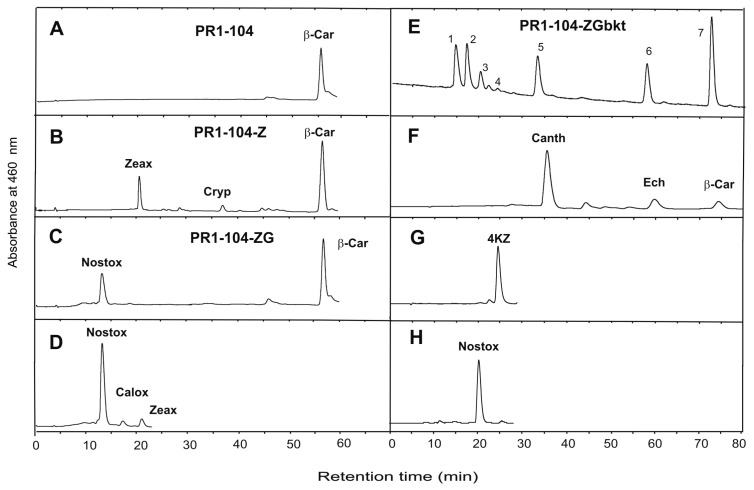
HPLC separation of hydroxy and keto carotenoids from *Xanthophyllomyces dendrorhous* lines obtained by consecutive transformation with different *trans* genes. Traces **A**–**D** separated in HPLC system I, **E**–**H** in system II. Standards are shown in traces **D**, **F**–**H**. Abbreviations (and assignment of corresponding peaks): β-Car β-carotene (7), Zeax zeaxanthin, Cryp β-cryptoxanthin, Nostox nostoxanthin (3), Calox caloxanthin, Canth canthaxanthin (5), Ech echinenone (6), 4KZ 4-keto-zeaxanthin (4).

**Figure 3:**
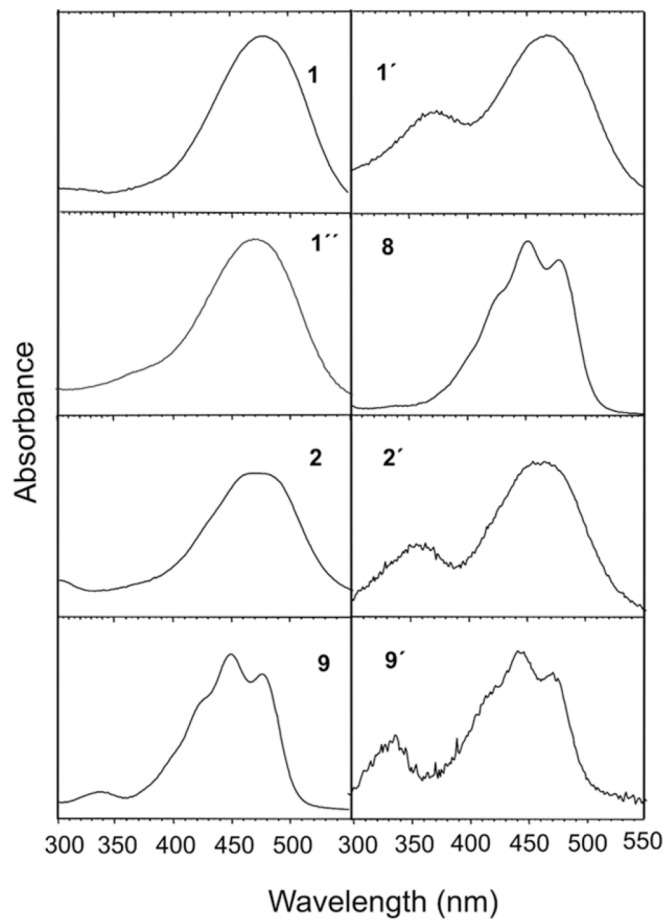
Absorbance spectra of hydroxy-keto carotenoids before (compounds **1** and **2** with *cis* isomers **1’, 1’’** and **2’**) and after reduction (compounds **8** and **9** with *cis* isomer **9’**).

Although fungi are in general unable to synthesize zeaxanthin or other hydroxy-carotenoids, formation of the 3,3’-dihydroxy-β-carotene can be engineered into *X. dendrorhous* [12]. This potential was extended for the synthesis of other derivatives with up to six hydroxyl groups. As outlined in Figure 1, this was achieved by a strategy involving the use of a β-carotene accumulating mutant of *X. dendrorhous*, its consecutive transformation with three microbial transgenes and finally the reduction of the 4- and 4’-keto groups. Figure 2 shows the HPLC analysis to identify some of the β-carotene-derived oxo compounds from the transgenic lines. Transformation with a bacterial 3-hydroxylase gene changed the PR1-104 from a β-carotene accumulator (trace A) to a transformant synthesizing zeaxanthin together with small amounts of the intermediate β-cryptoxanthin (trace B). In a second transformation step, with a bacterial 2-hydroxylase gene, the line PR1-104-ZG was obtained in which β-carotene was converted to nostoxanthin (trace C) as indicated by the nostoxanthin standard in trace D. Oxygenation of nostoxanthin was increased by a third transformation step with an algal 4-ketolase gene. In the resulting transformant PR1-104-ZGbkt, several carotenoids were detected. For base-line HPLC separation of these very polar oxo carotenoids, system I had to be changed to system II (Figure 2, trace E). In addition to the expected nostoxanthin-derived keto carotenoids, other carotenoids such as β-carotene (peak 7) and its keto derivatives echinenone (peak 6) and canthaxanthin (peak 5) as well as 4-keto-zeaxanthin (peak 4) and nostoxanthin (peak 3) were generated and could be identified with standard carotenoids (Figure 2, traces F and G). Compounds **1** and **2** of trace E are highly polar diketo and mono keto derivatives, respectively, as indicated by their optical absorbance spectra exhibiting a typical shape for keto carotenoids and an absorbance maximum of 475 or 468 nm (Figure 3).

**Figure 4:**
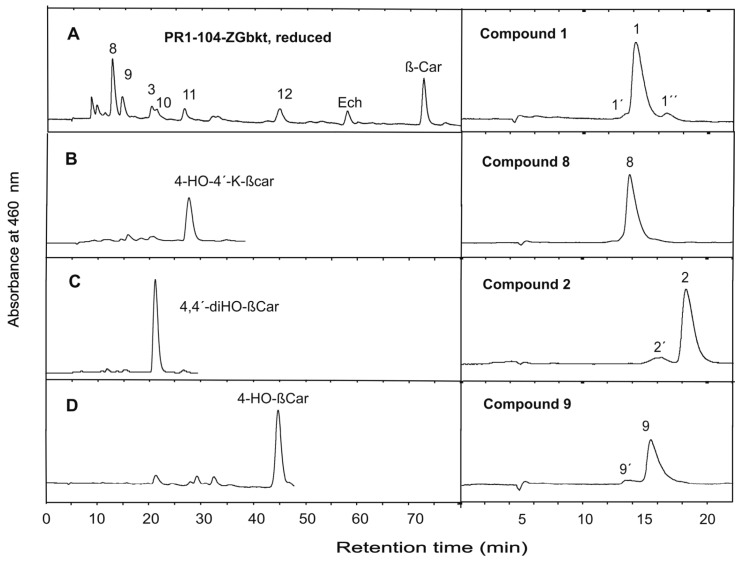
HPLC separation of hydroxy carotenoids after reduction (**A**–**D**) and isolated peaks 1 and 2 from Figure 2 and their reduction products in system II. Standards are shown in traces **B**–**D**. Abbreviations (and assignment of corresponding peaks): β-Car, β-carotene; Ech, echinenone; 4-HO-4’-K-βcar, 4-hydroxy-4’-keto-β-carotene (11); 4,4’-diHO-βCar, 4,4’-dihydroxy-β-carotene (10); 4-HO-β-Car, 4-hydroxy-β-carotene (12).

The carotenoid extract from transformant PR1-104-ZGbkt was reduced and separated by HPLC (Figure 4A). New peaks 8 to 12 emerged, which were not present in the non-reduced sample. Peaks 10 to 12 could be identified by chromatography of standards as 4,4’-dihydroxy-β-carotene (trace C), 4-hydroxy-4’-keto-β-carotene (trace B) and 4-hydroxy-β-carotene (trace D). Peaks 8 and 9 exhibited similar spectra with absorbance maxima and shoulder values at 424, 450 and 477 nm (Figure 3). Both compounds were obtained when isolated individual compounds from the non-reduced fraction were reduced. Isolated compound **1** changed into compound **8** and **2** into **9** (Figure 4, right part). The absorbance spectra of the all-*trans* isomers 1, 2, 8 and 9 together with individual *cis* carotenoids indicated with same primed number are shown in Figure 3.

**Figure 5:**
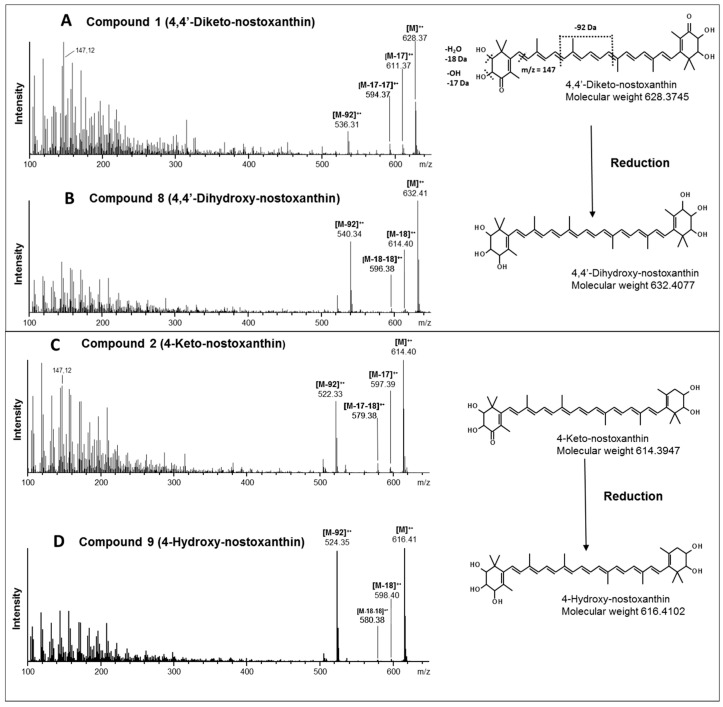
(**A**–**D**) MS-MS analysis of de-novo generated hydroxyl and keto-hydroxy β-carotene derivatives before and after reduction. The structures are indicated including the fragmentation pattern exemplified for 4-keto-nostoxanthin.

Final identification of compounds **1**, **2**, **8** and **9** was performed using high resolution mass spectrometry (Figure 5). Compound **1** showed a molecular mass of 628.3745 Da (Table 2) and fragments of M-17, M-17-17 and M-92 (Figure 5A). This molecular mass identifies compound **1** as 4,4’-diketo-nostoxanthin. Its reduction product, compound **8**, with a molecular mass of 632.4077 Da (Table 2) is regarded as 4,4’-dihydroxy-nostoxanthin. Instead of the M-17 and M-17-17 fragments of compound **1**, the fragments M-18 and M-18-18 were present in its mass spectrum. (Figure 5B). Therefore, compound **8** is regarded as 4,4’-dihydroxy-nostoxanthin. Compound **2** exhibits a fragmentation pattern in which the M-17-17 fragment of compound **1** is replaced by M-17-18 (Figure 5C). It has a molecular mass of 614.3947 Da (Table 2). This identifies compound **2** as 4-keto-nostoxanthin. Upon its reduction, the molecular mass increased to 616.4102 Da (Table 2). The M-92 fragment is retained but instead of M-17, an M-18 fragment and instead of M-17-18, an M-18-18 fragment appears (Figure 5D). These features of compound **9** correspond to 4-hydroxy-nostoxanthin. Common to the keto carotenoid **1** and **2** is a prominent peak at 147.12, which is much less pronounced in the reduced compounds **8** and **9**. The concentrations of these keto carotenoids in *X. dendrorhous* were determined as 37.4 ± 1.1 (µg/g dw) for 4,4’-diketo-nostoxanthin and 48.8 ± 1.4 (µg/g dw) for 4-keto-nostoxanthin. Their chemical reduction was almost complete (Figure 4A), which implies similar concentrations for 4,4’-dihydroxy-nostoxanthin and 4-hydroxy-nostoxanthin, respectively.

## 4. Discussion

Production of carotenoids by genetically engineered yeasts proved to be a promising alternative to chemical synthesis or extraction from plants [26]. The red yeast *X. dendrorhous* is the most versatile host with the highest carotenoid yield among fungi [5]. It is possible to construct and implement pathways to different carotenoid structures into this yeast. As an example, the synthesis to multi-oxygenated carotenoids was chosen in this publication (Figure 1) to demonstrate the potential of *X. dendrorhous* as a production platform for complex carotenoid structures. This was possible by extension of the pathway from accumulating β-carotene. The step-by-step transformation resulted in intermediary lines accumulating zeaxanthin or nostoxanthin as major carotenoids (Figure 2). Nostoxanthin is a carotenoid found in cyanobacteria [27] and was accumulated in recombinant *Escherichia coli* [14]. A final *X. dendrorhous* line PR1-104-ZGbkt transformed with three microbial genes produced 4-keto-nostoxanthin and 4,4’-diketo-nostoxanthin (Figure 2). Both carotenoids are extremely rare in nature and have been identified before only from two bacteria *Brevundimonas* SD212 and *Rhizobium lupine* [28,29]. By reduction of both keto carotenoids isolated from our line PR1-104-ZGbkt, the novel carotenoids 4-hydroxy-nostoxanthin and 4,4’-dihydroxy-nostoxanthin were obtained (Figure 4). Some of the isolated oxo carotenoids separated into several geometrical isomers on the C30 column (Figure 3). In each case, the all-*trans* isomer dominated. The isomers **1’** and **2’** showed a *cis* peak at 370 nm and **9’** at 330 nm. According to their height in relation to the dominating absorbance maximum, their position in front of the all-*trans* isomer on a C30 column and in comparison to astaxanthin for the keto derivatives [6] and to zeaxanthin for 4-hydroxy-nostoxanthin [30], these isomers are most likely 13-*cis*. In contrast, **1’’** without a *cis* peak may be a 9-*cis* 4,4’-diketo-nostoxanthin isomer.

The keto carotenoids and their reduction products were identified by high resolution mass spectrometry (Figure 5). For all of them, the correct molecular masses and the typical prominent fragments were obtained (Table 2). As indicated in the right part of Figure 5A, fragment M-92 originates from an in chain elimination of a toluene unit and is an indication of the central polyene chain [31]. All analyzed carotenoids show the elimination of hydroxyl groups either as water (M-18) or as neutral loss of 17 Da. In addition, an intensified peak was found at 147.12 Da in the spectra of the mono and diketo derivaties (Figure 5A,C). This is typical for a dehydrated 4-keto-β-ionone ring with cleavage of the C7,8 bond [31]. 

Our combined approach of mutant selection, genetic engineering and chemical modification is set as a general example of how novel carotenoids can be generated in *X. dendrorhous*. For the production of 4-keto-nostoxanthin and 4,4’-diketo-nostoxanthin, it is a proof of concept, which also indicates how to improve their yields in future studies. Judging from the relative low conversion of β -carotene to zeaxanthin (Figure 2B) and complete conversion of zeaxanthin to nostoxanthin (Figure 2C), the 3-hydroxylation step is regarded limiting in the formation of ketolated nostoxanthin. Formation of ketolated β-carotene derivatives echinenone and canthaxanthin demonstrate that the ketolation step is not limited (Figure 2E). It has previously been shown that conversion rates of transgenic reactions in *X. dendrorhous* are dependent on the number of *trans* gene copies integrated into the genome [32]. Therefore, either transformation with a plasmid carrying two copies of the 3-hydroxylase gene *crtZ* as demonstrated by Pollmann et al. [12] or repeated transformation with the *crtZ* gene is a promising way to improve intermediate conversion to the end product. In addition, overall carotenoid synthesis can be enhanced up to 3-fold by improvement of precursor supply in combination with increased flux into the carotenoid pathway [12] and in combination with a high carotenoids producing *X. dendrorhous* mutant, a total increase of carotenoid formation of up to 90-fold can be achieved [7].

## Figures and Tables

**Table 1:** Genes and oligonucleotides for PCR amplification of the *Xanthophyllomyces dendrorhous *transformation plasmids.

Plasmid	Primers
pPRcDNA1bkt830	β-Carotene 4-ketolase gene *bkt* from *Haematococcus pluvialis* (D45881)
	Primers: 5’-ATATGAATTCATGCACGTCGCATCGGCACT-3’ (forward) 5’-TATACTCGAGTCATGCCAAGGCAGGCACCAG-3’ (reverse)
pPR2TNH-crtG	β-Carotene 2-hydroxylase gene *crtG* from *Brevundimonas* SD212 [16] (AB181388)
	Primers: 5’-GGAATTCATGTTGAGGGATCTGCTCATC-3’ (forward) 5’-GCTCGAGTCACCGAAGAGGCGCTGAG-3’ (reverse)
pPR2TNo-crtZo	β-Carotene 3-hydroxylase gene *crtZ* from *Brevundimonas* SD212 [21]), codon optimized (KX063854)
	Sequences: 5’-GAATTCATGGCTTGGCTCACCTGGA-3’ (start) 5’-ATCTTCTTCTTCTGGAGCT TGAGTCGAC-3’ (end)

**Table 2:** Overview of identified multi-oxygenated nostoxanthins using UPLC-ESI-HRMS.

Compound	Trivial Name	Sum Formula [M]^+^	Calculated *m/z*	Detected *m/z*	Mass Error [Δppm]
**1**	4,4´-Diketo-nostoxanthin	C_40_H_52_O_6_	628.3758	628.3745	1.3
**2**	4-Keto-nostoxanthin	C_40_H_54_O_5_	614.3966	614.3947	1.9
**8**	4,4´-Dihydroxy-nostoxanthin	C_40_H_56_O_6_	632.4071	632.4077	0.6
**9**	4-Hydroxy-nostoxanthin	C_40_H_56_O_5_	616.4122	616.4102	2.0
